# Clinical challenges following early detection of ataxia telangiectasia through SCID newborn screening

**DOI:** 10.70962/jhi.20250052

**Published:** 2025-09-17

**Authors:** Thomas J. Weitering, Dagmar Berghuis, Maartje Blom, Michel A.A.P. Willemsen, Mirjam van der Burg

**Affiliations:** 1 Department of Pediatrics, Laboratory for Pediatric Immunology, Willem-Alexander Children’s Hospital, Leiden University Medical Center, Leiden, Netherlands; 2 Division of Pediatric Immunology and Stem Cell Transplantation, Department of Pediatrics, Willem-Alexander Children’s Hospital, Leiden University Medical Center, Leiden, Netherlands; 3 Department of Neurology – Pediatric Neurology, Radboud University Medical Center, Nijmegen, Netherlands

## Abstract

Ataxia telangiectasia (AT) is a rare autosomal recessive disease with progressive cerebellar neurodegeneration, variable degree of (combined) immunodeficiency, and increased risk of lymphoid malignancies. Especially patients with the hyper-IgM phenotype (HIGM-AT) are at risk for early-onset infections, cancer, and poor survival. AT can be a secondary finding in the T cell receptor excision circles (TRECs) based newborn screening for severe combined immunodeficiency, resulting in an early AT diagnosis, which poses challenges with respect to clinical management. It is yet unclear whether the positive TREC screening result is indicative for increased risk of the HIGM-AT phenotype. Here, we review the available literature on newborn screening outcomes and neonatal TREC levels in patients with AT. In addition, we discuss the available symptomatic treatment options for AT, including preemptive hematopoietic stem cell transplantation (HSCT), gene therapy, nicotinamide riboside (vitamin B3), and corticosteroids*.* Ultimately, we hope this article sparks international collaboration and prospective studies of newborn screening–identified patients with AT, focusing on outcomes and management.

## Teaser

Ataxia telangiectasia is a genetic disorder involving neurodegeneration, immunodeficiency, and cancer risk. It can be identified as incidental finding in newborn screening for SCID. Unfortunately, the clinical data and follow-up time are too limited, and the long-term effects on prognosis remain unclear, necessitating prospective studies.

## Introduction

Ataxia telangiectasia (AT, OMIM 208900) is a rare autosomal recessive multisystem disease named after its hallmark symptoms, namely progressive cerebellar ataxia and telangiectasias (1, 2). The affected gene, *ataxia telangiectasia mutated* (*ATM*) located at chromosome 11q22-23, encodes the ATM protein, which is often referred to as the master regulator of DNA double-stranded break repair processes (3). Other clinical manifestations in AT attributable to the role of ATM in DNA repair are variable combined (B and T cell) immunodeficiency, radiosensitivity, and increased susceptibility to (lymphoid) cancer (2, 3, 4, 5).

The immunodeficiency in patients with AT is variable but predominantly characterized by immunoglobulin (Ig) deficiency, affecting either or both IgG2 and IgA (2, 4, 5). We have recently shown that Ig deficiency in AT results from disturbed memory B cell development due to impaired class switch recombination (CSR), caused by altered DNA repair pathway utilization and changes in expression of germinal center genes (5). A small subgroup of patients with AT presents with normal to increased levels of serum IgM, in combination with decreased IgA and total IgG (5, 6, 7, 8). Prior to routine diagnostic genetics, these patients were sometimes misdiagnosed as hyper-IgM (HIGM) syndrome, especially before the onset of their neurological symptoms. In these patients, reduced naïve T cells and raised α-fetoprotein can be distinguishing factors for identifying AT (6). For the HIGM-AT diagnosis, there is no clear consensus on the timeframe and number of sampling time points in which these observations must be made. Most authors, however, agree that the observation should be present at least two distinct measurements throughout follow-up, including the most recent measurements. Patients with HIGM-AT have been shown to have increased mortality due to higher risks of infections and lymphoid malignancies (4, 5, 8). Additionally, Krauthammer et al. analyzed the effect of increased serum IgM on survival in a cohort of Israeli patients with AT (9). They also found reduced survival in the patients with AT with elevated IgM serum levels; however, the reduced survival was not correlated with an increased rate of malignancies. It remains challenging to compare all the available studies on the HIGM-AT phenotype due to the lack of consistent reporting and grouping of the patients. Based on the available data, we believe that concurrent presence of decreased serum total IgG and IgA, with either increased or normal IgM levels, is the best available sign of more deficient CSR and poor overall prognosis. Clinically, these Ig deficiencies and (sinopulmonary) infections in AT are often managed by either Ig-replacement therapies or antibiotics mostly targeting encapsulated bacteria (*Streptococcus pneumoniae* and *Haemophilus influenzae*). Amoxicillin, trimethoprim-sulfamethoxazole, and azithromycin are most often prescribed as prophylaxis to patients with AT. Additionally, vaccinations with the influenza and pneumococcal vaccines are often recommended to both patients and their inner circles (10).

In addition to the functional B cell impairment, patients with AT show distinct T cell subset abnormalities. Their T cell system is disbalanced, with specifically diminished numbers of peripheral blood naïve and recent thymic emigrant (RTE; CD4^+^CD31^+^) T cells (11, 12, 13). However, the memory T cell subsets are normal in their absolute counts and in their apparent function, because patients with AT typically do not suffer from opportunistic infections, which are normally caused by T cell dysfunction (2, 3, 7, 12). In newborns with AT, the number of RTE (naïve T cells) can be so low that some of these children can be identified coincidentally by newborn screening for severe combined immunodeficiency (NBS-SCID) (14, 15, 16). The NBS-SCID program is based on the quantification of T cell receptor excision circles (TRECs), which is a molecular biomarker for newly formed T cells (17). The NBS-SCID picks up a variety of neonatal conditions with low TRECs besides true SCID: leaky SCID and both syndromes and conditions with T cell impairment. Additionally, prematurity and low birth weight can cause low TRECs. In some cases, no clear cause is found (idiopathic T cell impairment), or the NBS-SCID is false positive (low TRECs but normal lymphocytes based on flow cytometric analysis). Consequently, AT can be a secondary finding in NBS-SCID, leading to identification of a subgroup of patients with AT being diagnosed much earlier in life, before onset of clinical symptoms (15). In the past, AT was typically only diagnosed after the onset of neurological symptoms at age of 2–3 years (2). Currently, there are no clear guidelines for clinicians on how to manage these early identified patients with AT, because the natural (immunological) course of the disease in this early phase is yet unknown. The early diagnosis also brings up the dilemma of preemptive allogeneic hematopoietic stem cell transplantation (HSCT) before the onset of (severe) recurrent infections and development of hematological malignancies.

In this clinical perspective, we address a triangle of immunological challenges in AT that are intertwined and originate from early identification of patients with AT as coincidental finding in TREC-based newborn screening programs. First, we will focus on which patients with AT are identified in NBS-SCID and whether these early identified patients have an increased risk for a worse clinical outcome. Secondly, we will focus on the patients with the HIGM-AT phenotype by better defining the immunological phenotype. Moreover, we question whether HIGM-AT co-occurs with more severely diminished T cells and TRECs to find a rationale for the more severe phenotype of this subgroup. In the third section, we discuss the current literature on preemptive and symptomatic treatments for AT, focusing on the potential of HSCT as a preemptive treatment.

## Early diagnosis of AT through NBS-SCID

### AT can be a secondary finding in TREC-based NBS-SCID

Newborn screening using TRECs is effective for diagnosing SCID in the first weeks after birth in often pre-symptomatic children, leading to improved outcomes after HSCT treatment or gene therapy (18). However, TREC-based screening identifies a plethora of different non-SCID T cell lymphopenia cases, with various causes and degrees of severity (15, 19, 20), including syndromes, reversible condition, and idiopathic causes of T cell impairment, as well as prematurity and low birth weight (17, 21). AT is among the unintentional, secondary findings of TREC-based screening, due to the unspecific nature of this screening approach. AT is not a target disease in NBS programs. Additionally, since the screening is not designed to detect AT, not all patients with AT will be identified through NBS-SCID.

### The ethical dilemma of AT as a secondary finding in NBS-SCID

The early diagnosis of AT as a secondary finding in NBS-SCID poses an ethical dilemma. The aim of NBS programs is to prevent morbidity and mortality from the targeted disorders through earlier treatment and with limited harm to unaffected infants. Non-actionable secondary findings raise concerns about the harm–benefit ratio of screening, and public health programs justifiably strive to prevent referral of these cases (22).

Non-actionable secondary findings may be relevant prognostically, but either effective treatments are not available or health benefits from early diagnosis are limited or uncertain. An early diagnosis of AT would prevent a long diagnostic process with emotional insecurity and anxiety for parents. In addition, as heterozygous carriers of a pathogenic ATM mutation have increased risk of developing cancer, early diagnosis of the newborn would allow early monitoring and screening for affected family members (2, 10). Genetic counseling and being informed about an increased risk for subsequent pregnancies would allow parents to make informed reproductive choices. However, these anticipated advantages are not embraced by the scope of a population screening program. In contrast, an accelerated diagnostic process of AT might prevent excessive invasive diagnostics, including ionizing radiation, and avoid life-attenuated vaccines, preventing malignancies and occurrence of serious infections by vaccine-strain organisms (23). An early diagnosis might therefore result in significant improved health outcomes for AT patients, but scientific evidence supporting this claim is lacking.

Apart from the potential benefits, an early diagnosis of AT could also provide a lot of psychological stress in the maturity period. Early diagnosis might prevent parents from enjoying the asymptomatic “golden years” and hamper with the child’s right to an open future. However, both parents of a child with AT as well as parents of healthy newborn counter these arguments, preferring an early diagnosis in the pre-symptomatic phase of the disease to prevent a long diagnostic process and to ensure optimal clinical guidance from the start (24, 25). Several other studies showed that parents are in favor of addition of childhood-onset disorders to NBS programs, as soon as a valid test is available, regardless of the actionability of the disease (26, 27, 28, 29). According to parents, the traditional aim of NBS programs is to identify infants with treatable conditions where early identification prevents irreversible health damage and creates a narrow scope for accessing all benefits of NBS. Parents believe that screening should identify actionable conditions, including (1) conditions where early interventions lead to health gain for the newborn, (2) conditions where early diagnosis avoids the lengthy diagnostic odyssey, and (3) conditions where parents will have reproductive options during subsequent pregnancies (30).

In NBS programs, clear information provision to parents is of utmost importance. Information should entail that SCID can be treated with HSCT, but other secondary findings might not have curative treatment options. Prior to genetic analysis, parents should be counseled by a clinical geneticist and pediatrician to inform them about severe disorders without highly effective treatment options such as AT.

### Newborn dried blood spot TREC levels in patients with AT

Previous publications have focused on the low TREC levels in patients with AT in the peripheral blood (31, 32, 33, 34). For example, Boyarchuk et al. show that 22 out of 25 patients with AT have severely reduced peripheral blood TRECs (31). Since AT is an incidental, secondary finding in NBS-SCID, we wonder what fraction of patients could potentially be diagnosed by the screening program. Four publications report on retrospectively measured TREC levels in dried blood spot (DBS) cards of patients with an established diagnosis of AT (14, 25, 35, 36). In the Swedish study by Borte et al., 4/4 patients with AT had TREC values in the (nonurgent) positive range (Table 1) (35). An urgent abnormal screening result implies a very low/absent TREC level requiring more rapid follow-up actions (21). Unfortunately, although Ig levels were measured and demonstrated IgA and IgG2 deficiency in 4/4 patients, detailed lymphocyte counts were not available. The authors described recurrent respiratory tract infections in 3/4 patients. Nourizadeh et al. retrospectively studied DBS cards of nine patients with AT (36). 9/9 of the patients with AT showed TREC values below the cutoff level, with 3/9 having near absent TRECs (i.e., urgent absent value) (36). Mallot et al. analyzed DBS cards of 13 patients with AT, of which 7 showed nonurgent positive results (14). All 13 patients with AT had a slight T lymphopenia at diagnosis with CD3^+^ counts below 1,500 per microliter, indicating a correlation between low neonatal TREC levels and decreased CD4^+^ T cell counts. Evaluation of clinical course did not point at a clear correlation between neonatal TREC levels and clinical outcome: frequencies of infections or malignancies did not differ between the groups with decreased and normal TREC levels. Additionally, in an earlier study by our research group, Schoenaker et al. performed TREC analysis on five AT DBS cards. Four out of five cards of these patients with AT showed low TRECs (25). Based on the fact that 24/31 (77.4%) of retrospectively analyzed newborn screening cards of patients with AT were below the cutoff values for an abnormal SCID screening result, we expect that the number of screening-identified AT cases will continue to increase as SCID newborn screening programs expand globally. Additional analyses are required to more precisely define phenotypical clinical and immune laboratory features associated with the low TREC values in these patients.

**Table 1.** Retrospectively analyzed DBSs of patients with AT

Number of analyzed AT DBS cards	TREC levels abnormal (below assay’s cutoff value [provided])	Other notable (clinical) findings	Reference
4	4/4 (15 copies per µl)	4/4 decreased IgG2 and IgA 3/4 recurrent pulmonary infections	(35)
9	9/9 (11 copies per 3.2 mm punch)	3/9 urgent abnormal value (1 TREC copy per 3.2 mm punch)	(36)
13	7/13 (25 copies per µL)	Lower CD4 T cells in patients with AT with reduced TRECs (R = 0.64)	(14)
5	4/5 low TRECs (NA)	NA	(25)
31	24/31 (77.4%) below respective assay’s TREC cutoff value		Totals

Urgent abnormal value indicates absent or very low TRECs without DNA amplification failure.

### Patients with AT identified through NBS-SCID

Next, we reviewed all published cases of early-diagnosed AT patients identified via NBS-SCID. To date, 15 newborns with an early diagnosis of AT identified after SCID newborn screening have been published (Table 2): five from California (USA) (14, 19), two from Wisconsin (USA) (37), one from New York (USA) (38), four from Canada (15), one from Germany (39), one from Sweden (40), and one from the Netherlands (16). In all of these cases, the TREC levels were low but in the nonurgent abnormal ranges of the respective screening cutoffs. The 2019 overview of the 2010–2017 screening results in California by Amatuni et al. showed that 5/213 (2.35%) of abnormal screening cases turned out to suffer from AT (19). The 2021 overview of Wisconsin, USA, reported two diagnosed cases of AT within 68 (2.94%) abnormal screening results. These two identified patients with AT had low CD3^+^ T cell counts of 1,474 and 945 cells per microliter. The 2017 Canadian overview of Mandola et al. reported that 15% of all identified cases with laboratory-confirmed immunodeficiencies were ultimately diagnosed with AT (4/26 patients) (15). This Canadian group was the only one to report on the immune parameters at a later time point of 8 mo of age. They report that all four patients with AT had sustained deficiencies of naïve T cells and B cells. Additionally, three out of four of the patients showed decreased T cell proliferation after PHA stimulation. Of particular importance, two out of their four patients with AT already presented with decreased IgA and total IgG at birth, in combination with increased IgM. These values did not recover after 8 mo follow-up and these patients could therefore be qualified as the HIGM-AT phenotype. They also showed diminished vaccination responses and were treated with Ig-replacement therapy. Regrettably, none of the other publications reported the longer-term immune parameters in detail, and all available reports are too recent to be able to report on longer-term outcomes. In the German publication on their newborn screening program by Speckmann et al., 1/88 (1.14%) of all abnormal results turned out to be AT (or 1/46 [2.17%] of all syndromic causes of low TRECs) (39). The recent 2024 New York case report by Lee et al. describes a NBS-SCID diagnosed patient with AT with severe T cell lymphopenia (counts CD3^+^ 407–879 cells/µl, CD4^+^ 277–556 cells/µl), low total IgG, low IgA, and normal IgM (i.e., HIGM-AT phenotype) (38). This patient required hospital admission for infection and subsequent treatment with Ig-RT and antibiotic prophylaxis with sulfamethoxazole-trimethoprim. Lastly, our group recently published a case report on a patient with AT who was identified via NBS-SCID (TREC levels in the nonurgent positive range), who presented with a SCID-like phenotype (counts CD3^+^ 300–600 cells/µl, CD4^+^ <200 cells/µl) and was started on antimicrobials (16). For this patient, preemptive HSCT was considered based on the HIGM-AT phenotype at diagnosis. However, HSCT was abandoned, because the T cells and IgM levels normalized within the first half year of life. The clinical dilemmas we faced in managing this patient were complicated by limited data on the natural progression of the immune parameters in the first years of life in patients with AT (15).

**Table 2.** Available published data on TREC-based NBS including number of identified patients with SCID and AT

Number of screened newborns	Number of identified SCID	Number of early identified AT	TREC levels, (patient; cutoff)	Other notable (clinical) findings	Reference(s)
3,252,156	50	5	5/5 abnormal (4–18; cutoff copies per µl)	5/213 (2.4%) abnormal screening results were AT Initial T cell counts of 800–1,323 cells per μL 1 patient presented with low IgG and IgA, normal IgM, and received Ig-RT	(19) (USA, California) (14)
670,580	8	2	2/2 abnormal (cutoff and TREC levels NA)	2/68 (3%) abnormal screening results were AT Initial CD3 counts were 1,474 and 945 cells per μL	(37) (USA, Wisconsin)
∼700,000	6	4	4/4 abnormal (7–13; cutoff 25 copies per µl)	4/63 (6.4%) of all abnormal screening results were AT 2/4 patients with AT showed increased IgM with decreased total IgG and IgA 3/4 patients with AT showed decreased T cell stimulation with PHA Diminished vaccination responses in AT	(15) (Canada)
58,834	1	1	Abnormal (5; cutoff copies per 3.2 mm punch)	1/64 (1.6%) recalls for secondary testing resulted in AT KRECs also below cutoff Patient asymptomatic at 1 year, but with IgA deficiency	(40) (Sweden)
1,900,00	25 SCID, 17 leaky SCID/Omenn syndrome	1	Abnormal (cutoff and TREC levels NA)	1/88 (1.1%) of total abnormal screening results was AT 1/46 (2.17%) of syndromic causes of low TRECs was AT Patient underwent successful, uneventful HSCT procedure at age 8 mo (alive and well at 18 mo of age)	(39) (Germany)
NA	NA	1	Abnormal (65; cutoff 200 copies per uL)	Low T cells, low IgG and IgA with normal IgM. Hospital admission for infection, broad-spectrum antibiotics, Ig-replacement therapy, and antibiotic prophylaxis	(38) (USA, New York)
NA	NA	1	Abnormal (8; cutoff 10 copies per 3.2 mm punch)	Low T cells with Pneumocystis jiroveci Pneumonia (PJP) prophylaxis (cotrimoxazol), HIGM, and decreased IgA that spontaneously improved	(16) (Netherlands)

**Figure 1.**
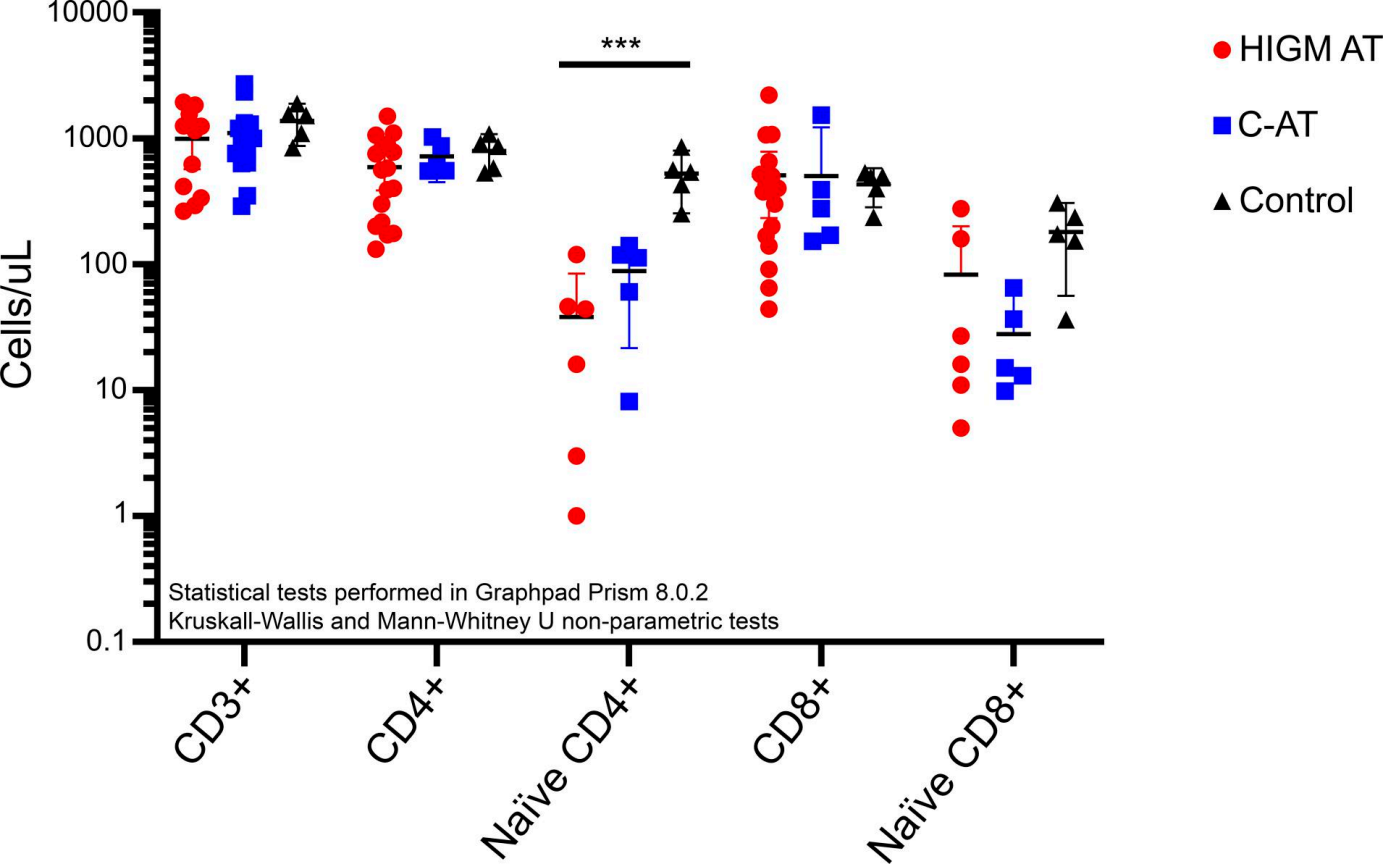
**T cell counts in patients with HIGM-AT compared to patients without HIGM-AT (classic AT [C-AT]) and healthy controls**. Counts are provided in cells/μl. The patient data are derived from multiple reports (6, 8, 12, 13, 16, 41, 42), with the counts provided in Table S1 (for HIGM-AT: *N* = 11 [CD3 counts], *N* = 17 [CD4/CD8 counts], *N* = 6 [naïve CD4/CD8 counts]; for C-AT: *N* = 12 [CD3 counts], *N* = 5 [naïve CD4/CD8 counts]). The healthy control (*N* = 5) counts are derived from Weitering et al. (2021) (12). Statistical testing was performed using GraphPad Prism, version 8.0.2. Kruskal–Wallis tests to compare all three groups and Mann–Whitney U tests to compare the HIGM-AT to the C-AT patients (*** = P-value <0.001).

It is still unknown whether low TRECs in AT are associated with higher frequency of the HIGM phenotype and worse clinical outcomes. Combining the published data on the T cell counts for the HIGM-AT and classic AT patients, no significant difference in the T cell counts were observed between the HIGM-AT phenotype patients and the other patients with AT (Fig. 1 and Table S1). Only the CD4^+^ naive T cell counts are significantly reduced in both the HIGM-AT and other AT patient groups compared with the controls. These data do not support the hypothesis of a more SCID with lower T cells in the HIGM-AT phenotype patients. However, it is important to initiate a future prospective international study to describe the natural course of AT early in life, especially focusing on immunological characteristics, their progression, and the long-term outcomes in these early-diagnosed AT patients.

## Early preemptive treatment options for AT: A role for HSCT?

### Debatable but potential role for HSCT as a preemptive treatment in AT

Patients with HIGM-AT have increased risk of early mortality due to recurrent pulmonary infections and lymphoid malignancies (5). The intrinsic genomic instability during V(D)J recombination and CSR puts patients with AT at increased risk of driving translocation events and tumorigenesis (3, 43). Furthermore, the T cell immunosurveillance against malignancies may be decreased. It has also been suggested that chronic inflammation plays a role in the neurodegeneration in AT (44, 45). Hence, there are many theoretical, potential benefits in replacing the immune system in patients with AT. These benefits have been shown in transplanted Atm^−/−^ mice models, showing sufficient engraftment, inhibition of thymic lymphoma formation, normalized T lymphocyte subsets, increased survival, and an improved ataxic phenotype (46, 47).

Despite both the theoretical benefits and benefits shown in animal models, HSCT remains a debatable treatment option for patients with AT (and other double-strand break [DSB] repair disorders) due to the potential toxicity from the conditioning chemotherapeutic regimen (48, 49). Currently, there exists published data on 15 patients with AT that have undergone HSCT, Table S2. In this limited number of transplanted patients, the outcomes are generally worse compared to other DNA-DSB repair syndromes, with nearly half (7/15) of the patients with AT dying after HSCT. There are, however, some important aspects to take into consideration. Despite the limited cohort size, there is a trend of worse outcomes in the patients that received myelo-ablative conditioning regimens, with 5/5 patients dying after HSCT (50, 51, 52, 53). These patients died from post-HSCT lymphoproliferative disease, (hepatic) organ failure, hemorrhagic cystitis, and pericardial effusion. In contrast, there is a trend for better outcomes when a modified Fanconi anemia reduced intensity conditioning (RIC) protocol was used (39, 47, 54, 55). 7/8 patients that survived received this conditioning regimen, of which at least four underwent a complication-free, uneventful HSCT procedure with adequate immune reconstitution. Some of the authors suggest these cases showed delayed onset and slower progression of neurodegenerative symptoms (4/8 surviving patients) (47, 54). They plead a potential role of HSCT as a preemptive treatment for patients with AT. By initiating treatment before onset of infections or malignancies, they argue increased odds of successful, uneventful transplantations. From a neurological point of view, infants diagnosed with AT through NBS-SCID would be ideal candidates for HSCT, since they do not yet suffer from clinical symptoms of neurodegeneration. In other words, if HSCT really has a positive effect on the neurodegenerative disorder of AT, it is reasonable to assume that early identified, pre-symptomatic children will benefit the most.

### Other promising upcoming treatments for AT

Unfortunately, due to progressive neurological degeneration in AT, there is no definitive curative treatment available (2). Although gene therapy has shown promising results in vitro and in mice (56)—and the first personalized (single case) studies are on their way—it is still a future prospect at best for larger groups of patients with AT. These approaches remain complicated by the large gene size of *ATM*, the multi-organ disease mechanisms, the necessity to cross the blood–brain barrier, as well as the fact that most patients have private mutations. Potentially, there may be a future role for gene editing approaches to correct for frameshift, nonsense, and missense mutations and allow some level of ATM gene expression (57).

Approaches that do not rely on gene therapy or editing focus on mitigating the deleterious effects of ATM deficiency. One of these approaches aims to reduce the oxidative stress and dysregulated NAD^+^ metabolism (58). After successful results in animal studies with anti-oxidative treatments using nicotinamide riboside (NR; NAD^+^ precursor also known as vitamin B3), a recent clinical trial by Veenhuis et al. has first shown its benefits in human patients (59). During treatment with NR, the patients with AT showed significantly improved ataxia scores and improvements in their serum IgG values, without adverse effects. This study was performed in mostly adolescent patients with AT, with an average age of 17.5 years. Earlier initiation of NR therapy in AT may result in slower progression of neurodegeneration, which is subject of further study. Similar results with NR have been demonstrated in a smaller series from Norway (60) and a single case report from Germany (61).

Another approach is using corticosteroids to reduce neuroinflammation in patients with AT. A recent paper summarized available evidence from the literature showing potential benefits of corticosteroids in AT and reported the results of a large phase three randomized, double-blind, placebo-controlled clinical trial (the ATTeST study [NCT02770807]) with intraerythrocyte dexamethasone sodium phosphate (62, *Preprint*). NR and corticosteroids could potentially be combined for synergistic beneficial effects on the neurological phenotype, the quality of life, and hopefully the longer-term outcomes. Finally, the potential benefits of small molecules (e.g., n-acetyl-leucine) have been reported (63), and the results of a large clinical trial with a dietary intervention (triheptanoate) are on their way (for review see Kuhn et al. [64]).

Taking everything into consideration, HSCT remains a debatable treatment option in AT. We do, however, hope to spark future prospective clinical trials in patients with AT that assess preemptive HSCT using RIC regimens with longer term follow-up and a focus on the potential neurological benefits. We believe that the HIGM-AT phenotype patients, with their deficient Ig-isotypes and increased risk of severe recurrent infections and malignancies and subsequent dramatically shortened lifespan, are the ideal inclusions for such a trial. Successful HSCT in patients with AT in early life could work synergistically with other beneficial treatments, such as the anti-oxidative vitamin B3 and corticosteroids. This raises hope for a future combination of treatments that may improve the outlook and quality of life for patients with AT.

## Conclusions and future perspectives

With the expansion of SCID newborn screening programs throughout the world, we expect to see an increase in early life, pre-symptomatically diagnosed patients with AT. We hope that this provides an opportunity to better chart the natural course of disease and immune parameters in the earliest years of life. For now, the consequences of being born with or showing severely reduced TRECs in patients with AT are unknown. As it stands, only the serum Ig phenotype and not the T lymphocyte levels of patients with AT has been linked to decreased survival (4, 5). Nonetheless, it remains a logical thought that having decreased T cells correlates with decreased class-switched Ig-isotype levels. While patients with AT with elevated IgM levels have been shown to have lower TRECs later in life (9), their neonatal TREC levels remain unknown. The HIGM-AT patients, however, do not seem to have a more significant naïve and total T lymphopenia compared to other patients with AT later in life. Prospective monitoring of patients with AT that are identified in the newborn screening programs for SCID will allow us to better assess the risk of developing the HIGM-AT, CSR-deficient phenotype with worse prognosis.

Besides clinical monitoring, Ig-replacement therapy, and antibiotic prophylaxis, three unfortunately non-curative, but disease-mitigating treatments for AT can currently be considered. The first option, HSCT, which replaces the diseased bone marrow to reduce the theoretical risk of lymphoid malignancies and infections, remains debatable in AT. We currently recommend HSCT for AT, mostly in the context of clinical trials, using a conditioning regimen based on the modified Fanconi anemia RIC protocol. Additionally, we believe preemptive, pre-symptomatic HSCT can be considered in patients with AT with a SCID-like immunodeficiency phenotype and a suitable donor. Long-term follow-up would be required to truly assess the benefits, including those for the neurological phenotype and its progression. The other two options, anti-oxidative NR (59) and corticosteroid (62, *Preprint*) treatments, have both been proven as safe and effective for reducing ataxia scores in AT. Perhaps there is a future role for a combined treatment with NR, corticosteroids, and/or HSCT in patients with AT. As a closing remark, we hope this article sparks international collaboration to start prospective studies of newborn screening-identified patients with AT, focusing on the outcomes and management.

## Online supplemental material

Table S1 shows T-cell subset peripheral blood counts of HIGM-AT patients, Classic AT and 5 healthy controls (plotted in Fig. 1). Table S2 shows the literature overview of HSCT in AT.

## Supplementary Material

Table S1 shows T-cell subset peripheral blood counts of HIGM-AT patients, Classic AT and 5 healthy controls (plotted in Fig. 1).

Table S2 shows the literature overview of HSCT in AT.
