# Supplementary material for: Clinical challenges following early detection of ataxia telangiectasia through SCID newborn screening
Source: J Hum Immun. 2025 Sep 17;1(4):e20250052. doi: 10.70962/jhi.20250052 (PMC13177395; doi:10.70962/jhi.20250052)
Supplement: Table S2 — shows the literature overview of HSCT in AT. [file jhi_20250052_tables2.docx]

**Supplemental Table 2 Literature overview of HSCT in AT**

| **Patient** | **Age at HSCT & Gender** | **Indication for HSCT** | **Year of HSCT** | **Donor, trans-plant type** | **Conditio-**  **ning type** | **Conditioning protocol** | **aGvHD** | **Follow-Up** | **Outcome** | **Reference** |
| --- | --- | --- | --- | --- | --- | --- | --- | --- | --- | --- |
| 1 | 13 years, M. | Malignancy (NHL; EBV-associated lymphoproliferative disease (EBV-LPD)) | 2014 | HLA-identical sibling, BM | RIC | Bu 1.6mg/kg  Flu 180 mg/m^2^  Cy 40mg/kg  RTX (2x 375 mg/m2) | No GvHD | 30 months.  Life-threatening toxicity in several organ systems due to conditioning:  Haemorrhagic cystitis, veno-occlusive disease, septicaemia, gastro-intestinal bleeding.  Good immune reconstitution.  Able to sit and stand without support; walks a few steps with assistance. | Alive | Slack 2018 (Ref. 50)  Recent systematic review; Sabino Pinho de Oliveira et al 2020 (Ref. 51)  Beier 2016 (Ref. 52) |
| 2 | 8 months, M. | Infection | NA | MUD, BM | MAC | Treo 36mg/m^2^  Flu 150 mg/m^2^  Campath 1mg/kg | Grade 1-2 skin | 6 months.  EBV-PTLD. | Dead | Slack 2018 (Ref. 50)  Registry data |
| 3 | 22 months, F. | Bone Marrow Failure | NA | Matched Family Donor, BM | MAC | Treo 46 mg/m^2  Flu 150 mg/m^2 | Grade 3, liver and skin | 20 months.  PTLD.  Hepatic failure | Dead | Slack 2018 (Ref. 50)  Registry data |
| 4 | 101 months, F. | NA | NA | Matched Sibling Donor, NA. | MAC | Bu (dose NA)  Cy (dose NA) | Grade 2, Gastro-intestinal tract and skin | 4 months.  Multi-organ failure. | Dead | Slack 2018 (Ref. 50)  Registry data |
| 5 | 138 months, M. | Malignancy | NA | Matched Family Donor, PBSC. | RIC | Flu (150 mg/ m^2^),  Cy (0.3 mg/kg). | Grade NA skin. | 11 months,  Extensive cGvHD skin.  Interstitial pneumonitis. | Dead | Slack 2018 (Ref. 50)  Registry data |
| 6 | 144 months, M. | Malignancy | NA | Matched Sibling Donor, BM. | MAC | Bu (dose NA), Cy (dose NA) | Grade 2 skin. | 3 months.  Pericardial effusion.  Hemorrhagic cystitis. | Dead | Slack 2018 (Ref. 50)  Registry data |
| 7 | 54 months, M. | T-ALL | NA | Matched Sibling Donor, BM. | RIC | Bu (2mg/kg)  Flu 150 mg/m^2^  ATG (80mg/kg),  OKT3 (anti-CD3, dose NA). | No GvHD | 48 months.  Haemorrhagic cystitis.  CMV reactivation. | Alive | Slack 2018 (Ref. 50)  Registry data |
| 8 | 22 months, M. | Hyper-IgM phenotype, with recurrent severe respiratory infections and hepato-splenomegaly. | NA | Matched Family Donor, BM. | MAC | Treo (3x 12 g/m2), Flu (5x 30 mg/m2), ATG-Fresenius (3x 20 mg/kg). | Grade 3 ( skin 2, liver 2, gut 0). | 32 months.  EBV-PTLD.  Fulminant hepatic failure with encephalopathy. | Dead | Slack 2018 (Ref. 50)  Ghosh 2012 (Ref. 53)  Recent systematic review; Sabino Pinho de Oliveira et al 2020 (Ref. 51) |
| 9 | 3 years, M. | T-ALL, high-risk chemotherapy with allo-HSCT. | 2009 | HLA-identical sibling, PBSC. | NA (chemo- therapy cycles,  followed by  conditioning) | Modified high-risk chemotherapy with subsequent allo-HSCT. ALL IC-BFM 2002 protocol, with adjustments based on Seidemann 2000 BFM experience (Ref. 65).  SCT conditioning:  Busilvex (0.5 mg/kg, 2 times/day; total dose 2 mg/kg)  Fludarabine (5x 30 mg/m2), ATG-Fresenius (20 mg/kg) | No GvHD | 9 years.  Complete leukemic/ hematological remission.  Good immune reconstitution.  Adenovirus treated with cidofovir.  EBV treated with rituximab.  Received 4x Donor Lymphocyte Infusion, due to poor donor chimerism.  HSCT did not worsen neurological status.  coordination deficits that make him wheelchair-bound. | Alive | Ussowicz 2018 (Ref. 66)  Recent systematic review; Sabino Pinho de Oliveira et al 2020 (Ref. 51) |
| 10 | 12 months, F. | Radiosensitive SCID, e.c.i. (No genetic diagnosis prior to HSCT). | NA | MUD 10/10, BM | RIC | Bu (2x 0.5 mg/kg/d), Flu (6x 30mg/m2), Cy (2x 20 mg/kg/d), Campath (1x 0.25 mg/kg and 3x 0.5 mg/kg/d)  GEFA03 protocol. | Steroid-responsve grade 1 skin GvHD. | 5 years.  Adequate  Coombs-positive hemolytic anemia treated with steroids.  Good immune reconstitution.  Neurological symptoms started after HSCT, and she was diagnosed with AT. | Alive | Ussowicz 2018 (Ref. 66)  Recent systematic review; Sabino Pinho de Oliveira et al 2020 (Ref. 51) |
| 11 | 23 months, M. | Pre-emptive – sibling with severe AT phenotype (early SCID with opportunistic infections, NHL complicated by HLH, died at age 6) | NA | MUD, BM | RIC | Bu (2x 0.5 mg/kg/d), Flu (6x 30mg/m^2^), Cy (2x 20 mg/kg/d)  Fanconi protocol | No GvHD. | 2 years.  Uneventful HSCT.  Adequate immune reconstitution, but with mixed chimerism.  Slow neurological deterioration. | Alive | Ussowicz 2018 (Ref. 66)  Recent systematic review; Sabino Pinho de Oliveira et al 2020 (Ref. 51) |
| 12 | 4 years, M. | Pre-emptive;  Mild recurrent airway infections.  Skin and joint granulomas. | 2012 | HLA-identical sibling, BM. | RIC | Flu (5x 30 mg/m2/d), Cy (4x 20 mg/kg/d),  ATG(20 mg/kg) | No GvHD. | 6 years.  Uneventful HSCT.  Adequate immune reconstitution.  Milder neurological deterioration. | Alive | Bakhtiar 2018 (Ref. 55)  Duecker 2019 (Ref. 47)  Recent systematic review; Sabino Pinho de Oliveira et al 2020 (Ref. 51) |
| 13 | 5 years, M. | Pre-emptive, mild recurrent airway infections | ? | HLA-identical sibling, BM. | RIC | (Flu (dose NA), Cy (dose NA), serotherapy (ATG?))  Presumably same doses as patient 12, but not specified. | No GvHD. | 1.5 years.  Uneventful HSCT.  Adequate immune reconstitution.  BK-virus and metapneumovirus intermittent viral reactivations.  Episode of pulmonary hypertension, responsive to therapy. | Alive | Duecker 2019 (Ref. 47) |
| 14 | 6 years, F. | Local destructive growth of granuloma, likely due to live-attenuated Rubella vaccination | ? | MUD 10/10, PBSC. | RIC | Flu (5x 30 mg/m2/d),  Cy (4x 30mg/kg/d), ATG Fresenius (3x 20 mg/kg/d).  Bahktiar’s 2018 modified Fanconi RIC regime. | Skin grade 3, steroid-responsive. | 6 months.  Granuloma resolution.  Mild viral reactivations (adenovirus and EBV).  Severe hepatic veno-occlusive disease. | Dead | Baumann 2022 (Ref. 67) |
| 15 | 8 months, gender NA | Newborn Screening Identified, Germany | 2022-2023 | ? | RIC (?) | Unknown | No GvHD | Clinically well at 18 months (10 months post-Tx) | Alive | Speckmann 2023 (Ref. 39) |

NA = not available.
